# Efficacy of the modified parallel method combined with the double-guide-wire technique for safer endoscopic ultrasound-guided hepaticogastrostomy

**DOI:** 10.1093/gastro/goaf048

**Published:** 2025-06-06

**Authors:** Koichiro Mandai, Takato Inoue

**Affiliations:** Department of Gastroenterology, Kyoto Second Red Cross Hospital, Kyoto City, Kyoto, Japan; Department of Gastroenterology, Kyoto Second Red Cross Hospital, Kyoto City, Kyoto, Japan

## Introduction

Endoscopic ultrasound-guided hepaticogastrostomy (EUS-HGS) is widely used; however, it may be associated with adverse events (AEs), such as stent migration, bleeding, bile leakage, and bile peritonitis [1]. Safer techniques are needed to improve patient outcomes and facilitate effective trainee education.

The double-guide-wire technique has been introduced to improve device-insertion support and facilitate stent placement, thereby reducing AEs [2, 3]. Additionally, bile aspiration (>10 mL) during EUS-HGS reduces post-procedural AEs [4]. During bile aspiration, guide-wire manipulation can be performed simultaneously by using the parallel method with an uneven double-lumen cannula (UDLC) (Piolax Medical, Kanagawa, Japan) [5].

However, the combined efficacy of these approaches in EUS-HGS remains unknown. We hypothesized that advancing guide wires further into the duodenal lumen would improve scope stability and facilitate stent insertion. To ensure guide-wire placement through biliary stricture into the duodenum, a modified parallel method was implemented. In February 2024, we introduced the PARACHUTE method—a modified parallel method combined with the double-guide-wire technique—to improve the safety of EUS-HGS.

## Methods

We retrospectively analysed a single-center case series to evaluate the efficacy of the PARACHUTE method in patients with malignant distal biliary obstruction (MDBO) undergoing EUS-HGS. This study was approved by the Institutional Review Board of the Kyoto Second Red Cross Hospital (IRB number: Sp2024-22). Written informed consent was obtained from all patients before the procedure.

**Figure 1.**
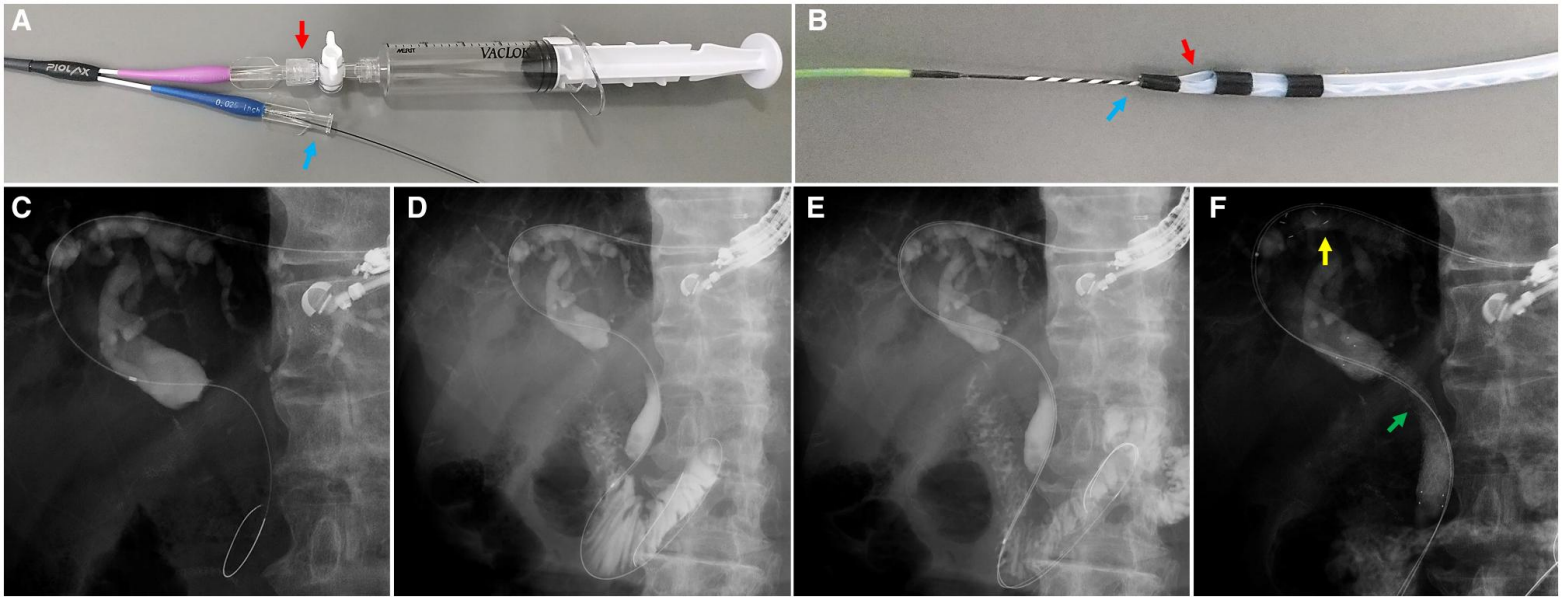
PARACHUTE method. (A) A locked suction syringe providing 20 mL of negative pressure is attached to the 0.035-inch proximal lumen of the UDLC (red arrow). (B) When the lock on the syringe is released, bile is aspirated automatically through the proximal lumen (red arrow). (A and B) Simultaneously, the guide wire is manipulated through the 0.025-inch distal lumen (blue arrow). (C) A 0.025-inch guide wire is manipulated through the distal lumen of the UDLC and advanced through the biliary stricture, while bile is simultaneously aspirated through the proximal lumen by the negative pressure generated upon release of the syringe lock. (D) After the guide wire and UDLC are advanced through the stricture into the duodenal lumen, contrast imaging is performed to evaluate the positional relationship between the stricture and the major papilla. (E) A second guide wire is inserted through the proximal lumen of the UDLC into the duodenal lumen to perform the double-guide-wire technique. (F) Antegrade stenting (green arrow) is performed across the stricture above the papilla and a transluminal drainage stent (yellow arrow) is placed by using the double-guide-wire technique.

A convex-type echoendoscope (GF-UCT260, Olympus Medical Systems, Tokyo, Japan) was used for all procedures. A 19-gauge needle (EZShot3Plus, Olympus Medical Systems, Tokyo, Japan) was used for puncture. After a 0.025-inch guide wire was inserted into the intrahepatic bile duct through the needle, the needle was exchanged for a short-type UDLC (tip length: 5 mm) over the guide wire and the modified parallel method was performed. While the original parallel method requires two assistants [5], our modified approach requires only one. A locked suction syringe with 20 mL of negative pressure was attached to the 0.035-inch proximal lumen of the UDLC (Figure 1A). Upon release, bile was automatically aspirated through the proximal lumen (Figure 1B), thereby eliminating the need for manual assistance. Simultaneously, the guide wire was advanced through the 0.025-inch distal lumen (Figure 1A and B) to access the duodenal lumen through the biliary stricture (Figure 1C). After the guide wire and UDLC were advanced through the stricture into the duodenal lumen by using the modified parallel method, contrast imaging was performed to measure the distance between the stricture and major papilla (Figure 1D). If the stent for the stricture was expected to extend across the papilla, then antegrade stenting was avoided to minimize the risk of pancreatitis. Regardless of antegrade stenting, another 0.025-inch guide wire was inserted through the proximal lumen of the UDLC into the duodenal lumen (Figure 1E). Stent placement was completed by using the double-guide-wire technique (Figure 1F). A partially or fully covered self-expandable metal stent (diameter × length: 8 mm × 12 cm) (Niti-S biliary S-type, TaeWoong Medical, Seoul, Korea; HANAROSTENT Biliary Full Cover Benefit, M.I. Tech, Seoul, Korea; or HANAROSTENT Biliary Partial Cover Benefit, M.I. Tech, Seoul, Korea) was placed from the left intrahepatic bile duct into the stomach.

Blood tests and plain computed tomography were performed to assess the stent position and monitor AEs on post-operative Day 1 and again between Days 5 and 7. Patients were observed in the hospital for ≥1 week post-operatively [6].

Operators who performed ≤33 or >40 EUS-HGS procedures were classified as trainees and experts, respectively [7]. Physicians who performed at least three of the following four steps were defined as operators: puncture and contrast injection; guide-wire placement; catheter or dilator exchange; and stent placement. Procedure time was defined as the duration from biliary puncture to scope removal.

Early AEs included procedure-related events occurring within 14 days [8]. AEs were evaluated according to the TOKYO Criteria 2024 and the severity grading system of the American Society for Gastrointestinal Endoscopy lexicon [8, 9].

## Results

Ten patients (median age: 76.5 years) with MDBO undergoing EUS-HGS using the PARACHUTE method at our institution between February 2024 and December 2024 were included. The most common primary disease was pancreatic cancer. EUS-HGS was successful in all patients, with antegrade stenting performed in one case. Trainees performed the procedure in 9 of the 10 patients. Segments B3 and B2 were punctured in seven and three patients, respectively. The mean diameter of the common bile duct above the stricture decreased from 15.8 mm before bile aspiration to 11.2 mm after aspiration. The median time from initiation of the parallel method to guide-wire insertion into the duodenum was 2.5 minutes. The caliber of the stent introducer was 5.9 Fr in eight patients and 8.5 Fr in two patients. Additional dilation of the puncture route was required in one case. The median procedure time was 29 minutes and no early AEs were observed (Supplementary Table 1).

## Discussion

The PARACHUTE method integrates the modified parallel method with the double-guide-wire technique and offers several advantages. It allows simultaneous bile aspiration and guide-wire manipulation, thus reducing the procedure time [5]. Additionally, it minimizes bile leakage. Although guide-wire insertion through a biliary stricture can be time-consuming, the method enables continuous and automatic bile aspiration during guide-wire manipulation, thereby reducing bile leakage into the peritoneal cavity. No cases of bile leakage or peritonitis were observed in this study. Aspiration-induced bile-duct decompression facilitates guide-wire insertion through the stricture [5]. In this study, guide-wire insertion into the duodenum was achieved rapidly in all patients. Furthermore, the double-guide-wire technique simplifies stent placement by improving scope stability [3]. Advancing both guide wires beyond the common bile duct into the duodenal lumen allows smoother and more controlled stent deployment. The modified parallel method may be effective for guide-wire insertion through strictures. Finally, the PARACHUTE method provides a recovery option when stent placement fails, as the second guide wire can be used for subsequent attempts.

Our findings suggest that the PARACHUTE method may improve the safety of EUS-HGS in patients with MDBO and facilitate trainee education. However, further multi-cohort studies are required to validate these findings.

## Supplementary Material

goaf048_Supplementary_Data
