# Supplementary material for: Efficacy of the modified parallel method combined with the double-guide-wire technique for safer endoscopic ultrasound-guided hepaticogastrostomy
Source: Gastroenterol Rep (Oxf). 2025 Jun 6;13:goaf048. doi: 10.1093/gastro/goaf048 (PMC12145175; doi:10.1093/gastro/goaf048)
Supplement: goaf048_Supplementary_Data [file goaf048_supplementary_data.zip › Supplementary Table 1.docx]

| **Supplementary Table 1.** Characteristics of patients treated with the PARACHUTE method and their outcomes | | | | | | | | | | | | | | | |
| --- | --- | --- | --- | --- | --- | --- | --- | --- | --- | --- | --- | --- | --- | --- | --- |
| Case | Age/Sex | Causes of MDBO | SAA | Concomitant cholangitis | Procedure type | Operator | Needle | Punctured BD (diameter, mm) | Diameter of CBD above the stricture, mm | | Time from parallel method to guidewire insertion in the duodenum, min | Additional dilation for the Puncture Route | Type of HGS stent | Procedure time, min | AEs ≤14 days |
|  |  |  |  |  |  |  |  |  | Before bile aspiration | After bile aspiration |  |  |  |  |  |
| 1 | 82/F | PC | No | No | HGS | Expert | 19 G | B3 (3.3) | 17.9 | 15.1 | 6 | None | FCSEMS | 16 | None |
| 2 | 77/M | PC | No | No | HGS | Trainee | 19 G | B3 (9.2) | 17.8 | 13.5 | 9 | Bougie | FCSEMS | 59 | None |
| 3 | 76/F | PC | No | No | HGS | Trainee | 19 G | B3 (8.3) | 10.2 | 9.1 | 3 | None | FCSEMS | 28 | None |
| 4 | 76/F | PC | No | Yes | HGS | Trainee | 19 G | B3 (9.0) | 12.5 | 9.1 | 2 | None | FCSEMS | 40 | None |
| 5 | 76/F | PC | No | No | HGS | Trainee | 19 G | B2 (4.2) | 24.4 | 15.7 | 4 | None | FCSEMS | 31 | None |
| 6 | 67/M | PC | No | No | HGS | Trainee | 19 G | B3 (3.3) | 13.5 | 8.8 | 2 | None | FCSEMS | 36 | None |
| 7 | 72/F | PC | No | No | HGS | Trainee | 19 G | B2 (4.0) | 16.2 | 9.4 | 2 | None | FCSEMS | 28 | None |
| 8 | 84/M | GC recurrence | DG | No | HGAS | Trainee | 19 G | B2 (4.9) | 17.5 | 14.7 | 3 | None | PCSEMS | 30 | None |
| 9 | 79/F | PC | No | No | HGS | Trainee | 19 G | B3 (5.7) | 10.4 | 7.4 | 1 | None | PCSEMS | 14 | None |
| 10 | 94/M | PC | No | No | HGS | Trainee | 19 G | B3 (6.1) | 17.8 | 9.5 | 1 | None | PCSEMS | 19 | None |
| AEs, adverse events; BD, bile duct; CBD, common bile duct; DG, distal gastrectomy; F, female; FCSEMS, fully covered self-expandable metallic stent; GC, gastric cancer; HGS, hepaticogastrostomy; HGAS, hepaticogastrostomy with antegrade stenting; M, male; MDBO, malignant distal biliary obstruction; PC, pancreatic cancer; PCSEMS, partially covered self-expandable metallic stent; SAA, surgically altered anatomy. | | | | | | | | | | | | | | | |
